# Telerehabilitation Approaches for People with Chronic Heart Failure: A Systematic Review and Meta-Analysis

**DOI:** 10.3390/jcm12010064

**Published:** 2022-12-21

**Authors:** Sara Isernia, Chiara Pagliari, Nuccia Morici, Anastasia Toccafondi, Paolo Innocente Banfi, Federica Rossetto, Francesca Borgnis, Monica Tavanelli, Lorenzo Brambilla, Francesca Baglio

**Affiliations:** 1 IRCCS Fondazione Don Carlo Gnocchi ONLUS, 20148 Milan, Italy; 2 IRCCS Fondazione Don Carlo Gnocchi ONLUS, 50143 Florence, Italy

**Keywords:** telerehabilitation, chronic heart failure, continuity of care, digital medicine, cardiology, rehabilitation

## Abstract

Introduction: Telerehabilitation (TR) for chronic heart failure (CHF) allows for overcoming distance barriers and reducing exacerbations. However, little is known about TR descriptors, components, and efficacy in CHF. Methods: This work systematically reviewed the TR strategies of randomized controlled trials in people with CHF. A meta-analysis was run to test its effect on exercise capacity and quality of life compared to no rehabilitation (NI) and conventional intervention (CI). Results: Out of 6168 studies, 11 were eligible for the systematic review, and 8 for the meta-analysis. TR intervention was individual and multidimensional, with a frequency varying from 2 to 5 times per 8–12 weeks. The TR components mainly included an asynchronous model, monitoring/assessment, decision, and offline feedback. A few studies provided a comprehensive technological kit. Minimal adverse events and high adherence were reported. A large effect of TR compared to NI and a non-inferiority effect compared to CI was registered on exercise capacity, but no effects of TR compared to NI and CI on quality of life were observed. Conclusions: TR for people with CHF adopted established effective strategies. Future interventions may identify the precise TR dose for CHF, technological requirements, and engagement components affecting the patient’s quality of life.

## 1. Introduction

Heart failure (HF) is an age-related global epidemic with a prevalence of over 37 million individuals worldwide [1]. Although significant steps forward have been gained by pharmacological therapies, people with HF may experience frailty and disability, and face a high risk of exacerbation and re-hospitalization after the acute condition. Especially the typical chronicity of HF, chronic heart failure (CHF), needs a comprehensive approach to prevent exacerbations, including a structured physical activity program to increase exercise tolerance and quality of life and reduce the probability of hospitalization [2,3]. In fact, center-based cardiovascular rehabilitation is recommended for CHF, generally consisting of weekly 60–90 min over 12–36 weeks of in-person sessions, which has been demonstrated to be effective in reducing hospital readmission rate and mortality [4,5]. Unfortunately, a standard center-based rehabilitation program may not be easily accessible to a consistent group of patients due to distance barriers and related costs, work, family commitments, and heavy pharmaceutical interventions [6]. To overcome this issue, home-based physical activity interventions have been proposed to manage CHF after discharge from the clinic with a non-inferiority efficacy than center-based treatments [7]. However, these treatments lack clinicians’ supervision and monitoring, which may be critical to the intervention’s adherence and effectiveness. Moreover, after hospital-based recovery, often HF patients are not prepared to be independent in autonomously managing prescribed cardiovascular rehabilitation at home, and the positive effects of the rehabilitation reduce over time [3,8].

Technological breakthroughs have prompted innovative telerehabilitation (TR) solutions by offering the possibility to deliver interventions at a distance, allowing clinic-home communication [9,10]. This approach introduces a new model of intervention in which both the clinicians and the patient are engaged in the care: the clinicians prescribe, adapt, and manage the treatment at a distance through healthcare platforms, and the patient attends and is empowered in the rehabilitation program by using the technological kit (e.g., platform, devices, digital contents) in his/her everyday living context. In detail, the clinician can communicate with the patient at home in a synchronous modality, adopting a TR synchronous model and using a videoconference system allowing him to mimic the face-to-face practice in the clinical setting, or in an asynchronous modality, according to the TR asynchronous model by using complex platforms allowing the decoupling of the communication flow and overcoming the 1:1 (1 clinician:1 patient) perspective [11]. Besides the TR model, the presence of monitoring/assessment of the treatment, decision, and feedback types are additional TR strategies. The intervention’s adaptability over time requires the therapist to monitor and assess the patient’s TR activity to make decisions and set the dose and intensity of the sessions. Also, the feedback provided by the therapist to the patients to report the progression of the rehabilitation program can be online during the course of the intervention, or offline at the end of the program. Without these actions, the rehabilitation program is not personalized and adapted over time and may not be considered comparable to a hospital-based treatment [12]. A recent meta-analysis revealed the TR effectiveness on functional capacity, peak oxygen uptake, and quality of life in people with HF. However, this contribution included heterogeneous studies, not detailing the care model adopted and comparing the effect of TR only with usual care, without distinguishing between no intervention versus conventional intervention in the hospital [13]. Therefore, evidence of the TR intervention effect specifically on CHF is still sparse, and little is known about the TR model adopted (synchronous/asynchronous model, assessment/monitoring, decision, online/offline feedback).

To fill this gap, the present study aims (i) to profile the TR for CHF in terms of descriptors of intervention (FITT: Frequency, Intensity, Type, Time), TR approach (model, assessment/monitoring, decision, and feedback), and adopted technologies (platforms, devices, digital contents), safety and adherence (systematic review), and (ii) to test the TR effect on two main cardiovascular primary outcomes in literature (functional capacity and quality of life) compared to no intervention (NI) and hospital-based treatments (CI) (meta-analysis).

## 2. Materials and Methods

A systematic review and meta-analysis were performed following the PRISMA (Preferred Reporting Items for Systematic Reviews and Meta-Analyses) guidelines [14]. This work has been registered and published in PROSPERO (CRD42022316822).

### 2.1. Eligibility Criteria

**Figure 1:**
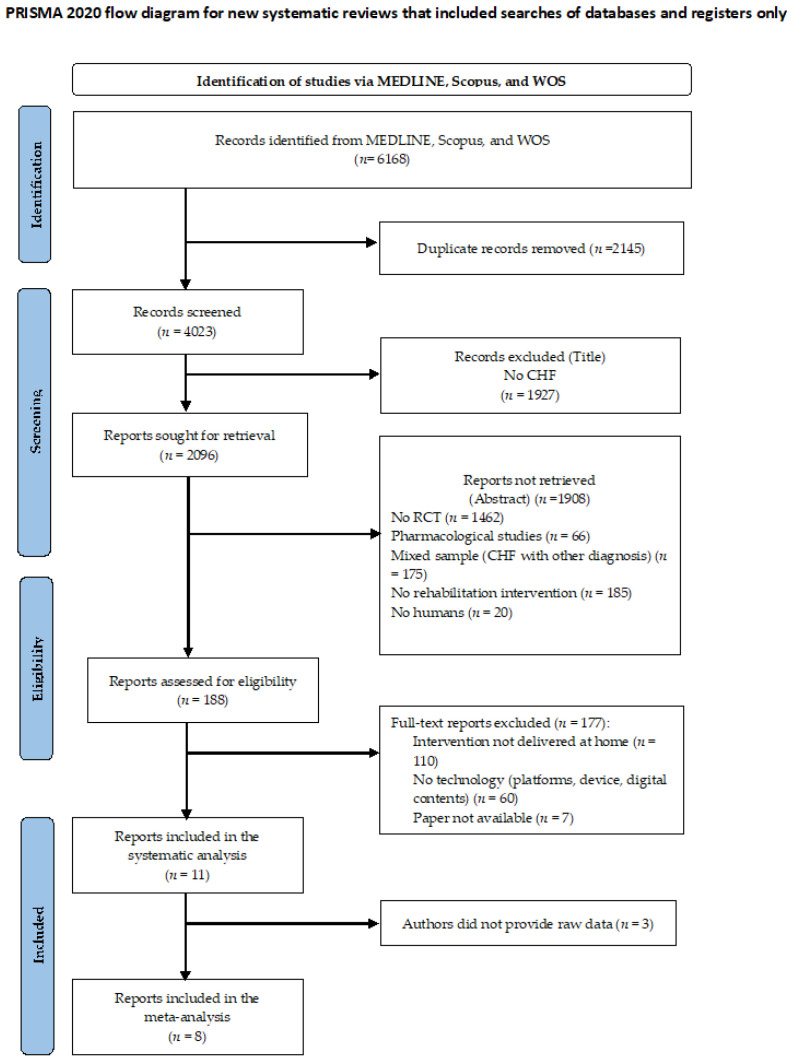
CONSORT Flow Diagram.

Studies were judged eligible to be included in the systematic review according to the following criteria: (i) primary study; (ii) with an RCT design; (iii) testing the effect of TR; (iv) in people with CHF. In addition, the pilot study design, delivery of TR out of the patient’s home, and the absence of physical activity in the TR program were considered exclusion criteria. The studies’ eligibility was screened in three steps: title, abstract, and full-text reading (see the Flow Diagram, Figure 1).

### 2.2. Interventions

Telerehabilitation: we referred to the TR as home-based treatments in which the rehabilitation was delivered at a distance by maintaining the communication between the clinician and the patient through technological facilities (platform, devices, etc.). This communication may provide feedback on the rehabilitation activity to the patient and the adaptability of the treatment along the TR period based on the progress gained by the patient. The presence of communication between the clinician and the patient’s home was considered fundamental since in the lack of clinic-patient interaction, the treatment lacks a fundamental rehabilitative component [12]. We considered telerehabilitation including physical activities and eventually combined educational, psychosocial, nutritional, and motivational intervention.

Comparators: two comparators were separately considered: no intervention, such as a condition without specific rehabilitation prescription but with a stable medication regiment and regular follow-ups (NI); and the conventional intervention (CI) condition, a rehabilitation program in the clinic combined with a stable medication regiment and regular follow-ups (hospital-based).

### 2.3. Information Sources and Study Selection

For the systematic review, the data bench utilized for the literature search were MEDLINE, Scopus, and Web of Science. The search was performed on 27 October 2022 using the following string: (((((((((Tele * OR virtual reality *) OR computerized treatment *) OR computerized training *) OR computer-assisted rehab *) OR serious game *) OR videogame *) OR home-based treatment *) OR home-based training *) AND chronic heart failure *). All studies published from 2010 to March 2022 were included in the literature search. No limitation concerning the language of publication was applied.

### 2.4. Selection and Data Collection Process

The eligibility screening was conducted blindly and independently by two researchers with an automatized software to speed and enhance the accuracy and reliability of the systematic review process (Rayyan platform [15]). Inter-reviewer discrepancies were solved by discussion to reach a consensus or by a third reviewer when an agreement was not reached. After the study screening and selection, data collection was conducted independently and blindly by the two researchers following a standardized form. Data collection focused on demographics and clinical characteristics of the sample (sex, age, inclusion and exclusion criteria), treatment FITT (frequency, intensity, time, type), TR actions (model, assessment/monitoring, decision, feedback), and technology used (TR platform, devices, digital contents). Finally, the studies’ patient-relevant and medical benefit outcome measures were collected. Also, for data collection, the inter-reviewer disagreements were solved either by consensus or by the third reviewer when an agreement could not be reached.

### 2.5. Study Risk of Bias Assessment

The “Tool for the assessment of study quality and reporting in the exercise” scale (TESTEX; [16]) was blindly utilized by the two reviewers to evaluate the quality of RCT studies. Eventual inter-reviewer disagreements were solved either by consensus or by the third reviewer. Each study selected for the systematic review was assessed based on 12 external and internal validity criteria, for a total score ranging from 0 to 15 (score equal to/lower than 7 = low-quality study; score comprised between 7–11 = good quality study; score higher than 11 = high-quality study).

### 2.6. Statistical Analysis

Statistical analyses were computed using RStudio, version 3, adopting the metafor R package. Exercise capacity and quality of life were considered fundamental outcome measures for CHF and included in the meta-analyses. First, the overall effect of TR on exercise capacity (both VO_2_ peak and 6 meter walk distance) and quality of life was computed. The unit of analysis considered was the standardized mean difference (SMD) of change from pre-treatment to post-treatment between TR and the control group. SMD as Hedges’ *g* and 95% confidence intervals (95% CI) were computed for outcomes of each study selected for the meta-analysis. The overall effect of TR on the specific outcome was pooled using a random-effect model. Corrections for inter-correlation among outcomes were assumed at 0 and 0.5. A *g* value ≤ 0.30, >0.30, ≥0.60 was interpreted as a small, moderate, and high effect size, respectively, according to Higgins et al. [17]. The proportion of true variance from total observed variance was reported by I^2^ statistic with 95% CI (an I^2^ value of 25%, 50%, and 75% suggested a low, moderate, and a high proportion of variance, respectively). Potential publication bias was investigated by reporting the funnel plot to detect eventual asymmetry and small study effect, and the estimated number of missing studies was checked using the trim-and-fill procedure. Cochrane’s recommendations were strictly followed to overcome limitations related to missing values.

## 3. Results

### 3.1. Study Selection

In total, 6168 studies were initially identified. Among these, 11 works were eligible for the systematic review, and 8 were included in the meta-analyses. The third reviewer was consulted twice to solve disagreements. Figure 1 reports the PRISMA flow diagram.

### 3.2. Risk of Bias in Studies

Three trials presented a high level of quality, six a good level, and two a low level of quality based on the TESTEX score (Table 1).

### 3.3. Participants

Table 2 reports the demographic and clinical characteristics of TR, NI, and CI of the trials selected for the systematic review. This review comprised a total of 1500 people with CHF. Among these, 782 patients underwent TR at home (650 males, mean age = 60.20 ± 5.38), 598 were administered an active comparator (CI; 505 males, mean age = 60.59 ± 3.81), and 120 followed no intervention (NI; 97 males, mean age = 66.60 ± 4.46). All the studies included a mixed etiology, both ischemic and non-ischemic. The left ejection fraction was reduced in most cases. The inclusion and exclusion criteria of each study included in the review are reported in Supplementary Material (Table S1).

### 3.4. Telerehabilitation Intervention Descriptors

Nine trials adopted individual rehabilitation sessions type [18,20,22,23,24,25,26,27,28], one study provided group sessions [19], and one study adopted a mixed approach [21]. Seven trials provided a multidimensional program including educational, pulmonary, and psychological interventions together with motor activity [18,19,20,21,24,25,28], while the rest of the studies provided a unidimensional motor treatment [22,23,26,27]. The treatment dose was heterogeneous among studies. The frequency of the program varied 2 to 5 times per week, with a median frequency of 5 times per week. The intensity was set based on the heart reserve in seven studies (about 40–80%; [18,21,22,23,24,25,27], but also in the Borg scale (score 9–13) in 4 cases [20,22,23,25]. One trial [19] used the perceived exertion scale, while another study [26] considered the VO_2_ peak. The treatment duration ranged from a minimum of 8 to a maximum of 12 weeks, with a median of 8 weeks. The time of each session lasted about 45 min (median), ranging from 30 to 60 min. Details on the FITT descriptors of TR intervention are reported in Table 3.

### 3.5. Telerehabilitation Actions

Model: Nine trials (82%) adopted an asynchronous TR model [18,20,22,23,24,25,26,27,28]. Two studies utilized synchronous monitoring [19,21].

Assessment and Monitoring: all trials assessed and monitored the patient’s rehabilitation performance during the intervention program by using videoconference software [19,21], wearable devices [18,19,20,26,27], an ECG device [22,23,24,25,28], or pedometer [26].

Decision: all the studies provided decisions during the intervention and modified the dose of the treatment during the program period.

Feedback: eight trials adopting the asynchronous TR model (89%; [18,22,23,24,25,26,27,28]) provided offline feedback, while one study [20] adopted a mixed approach.

Table 3 summarizes the TR approach of the studies included in the review.

### 3.6. Telerehabilitation Technology

Of the 11 trials selected in the review, only one study [19] (9%) presented a comprehensive technological kit fitted with a TR platform system, devices, and digital contents. Five studies [22,23,24,25,28] (45%) provided a technological kit including devices and a platform system. Two works [18,20] (18%) used monitoring devices and digital contents, two works [26,27] provided only monitoring devices, and one work [21] used only the platform system.

### 3.7. Telerehabilitation Adherence and Safety

Six studies [19,20,22,24,25,26] evaluated adherence to the TR treatment. Overall, the studies reported the participation rate in terms of the number of participants who adhered to the TR program. The average adherence rate was 86.07%. Similarly, four contributions [19,20,24,25] registered adverse events during the training period. Overall, the studies reported minimal adverse events. In one study [19], the adverse events were 1.56% during exercise training and 1.04% immediately after the training. One trial [25] reported three episodes of paroxysmal atrial fibrillation; one trial [19] highlighted an equal rate of adverse events in the TR and the NI group (three anginas, three diaphoreses, and two palpitation episodes). Nagatomi et al. [20] reported no serious adverse events (in the CI, one pyelonephritis, one gastrointestinal bleed, one pacemaker battery change, one wamble, one musculoskeletal pain, one emesis, one numbness; in the TR group, one heart failure, one anemia, one driveline infection, two chest symptoms, three musculoskeletal pain) and three readmissions in the TR and the CI group, not causally related to the intervention.

### 3.8. Efficacy of Telerehabilitation

Outcome measures evaluated in each trial are described in Table 4.

#### 3.8.1. Functional Capacity

**Figure 2:**
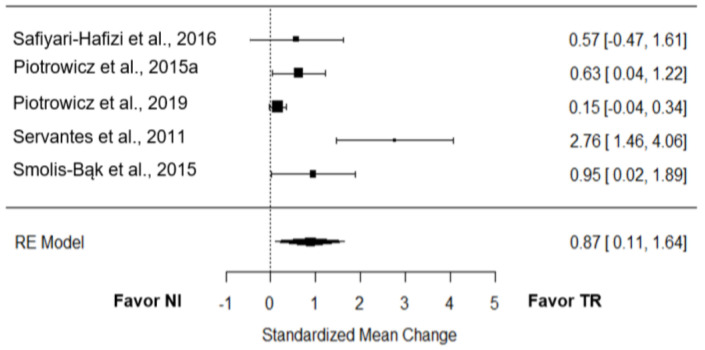
Effect of TR on VO_2_ peak compared to NI [22,24,26,27,28].

*VO_2_ peak:* Five studies [22,24,26,27,28] compared the effect of TR on VO_2_ peak to NI. The overall effect of TR on the VO_2_ peak was large and significant (*g* = 0.87; 95% CI = 0.11 to 1.64; *p* = 0.01; Figure 2). True heterogeneity across studies was large (I^2^ = 84.52%; Q = 19.45; df = 4; *p* < 0.01), and the funnel plot showed symmetry (SE = 1.43) (Supplementary Materials, Figure S1).

Only one trial provided raw data to test the effect of TR on VO_2_ compared to CI [25], with a small effect size (*g* = 0.21; 95% CI = −0.32 to 0.74). 

**Figure 3:**
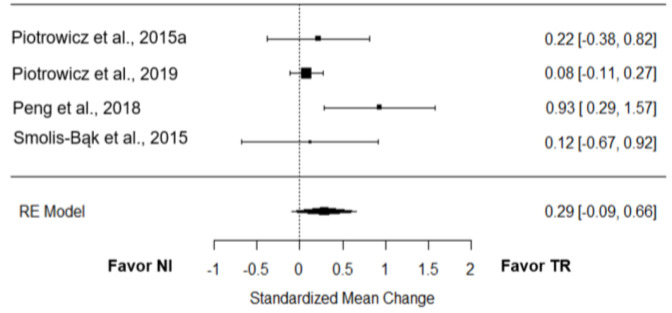
Effect of TR on the 6MWD compared to NI [21,22,24,28].

*6-minute walk distance:* Four studies [21,22,24,28] verified the effect of TR on the 6-minute walk distance (6MWD) performance compared to NI. The overall effect was small and non-significant (*g* = 0.29; 95% CI = −0.09 to 0.66; *p* = 0.13; Figure 3). True heterogeneity across the studies was moderate (I^2^ = 52.53%; Q = 6.25; df = 3; *p =* 0.10), and the funnel plot was symmetrical (SE = 1.38; Supplementary Materials, Figure S1).

**Figure 4:**
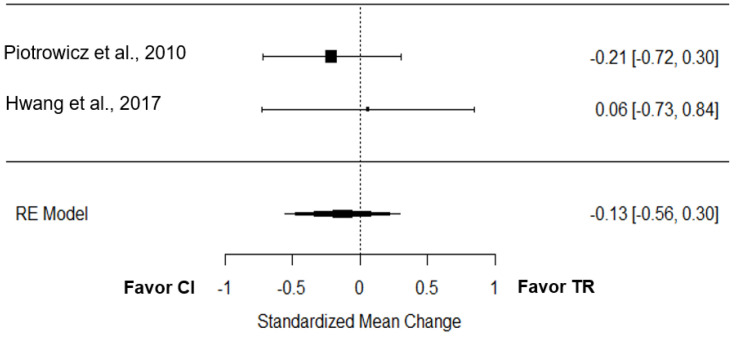
Effect of TR on the 6MWD compared to CI [19,25].

Two studies tested the effect of TR on the 6-minute walk distance performance compared to CI [19,25]. The overall effect was low and non-significant (*g* = −0.13; 95% CI = −0.56 to 0.30; *p* = 0.58; Figure 4). True heterogeneity across the studies was null (I^2^ = 0.00%; Q = 0.31; df = 1; *p =* 0.58), and the funnel plot was symmetrical (Supplementary Materials, Figure S1).

#### 3.8.2. Quality of Life

**Figure 5:**
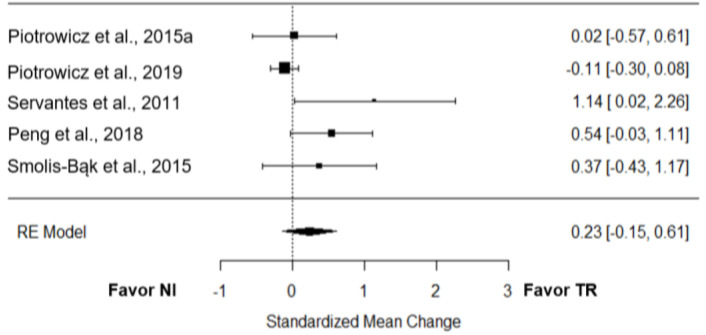
Effect of TR on QoL compared to NI [21,22,24,27,28].

Five trials [21,22,24,27,28] investigated the effect of TR on quality of life compared to NI. The overall effect was low and non-significant (*g* = 0.23; 95% CI = −0.15 to 0.61; *p* = 0.23; Figure 5). True heterogeneity across the studies was medium (I^2^ = 55.71%; Q = 9.61; df = 4; *p* = 0.05), the funnel plot was asymmetrical, and estimated 3 missing studies on the left side (SE = 1.48; Supplementary Materials, Figure S1). 

**Figure 6:**
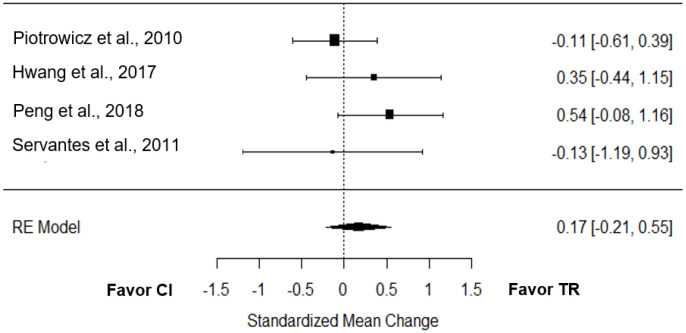
Effect of TR on QoL compared to CI [19,21,25,27].

Four studies tested the effect of TR on quality of life compared to CI [19,21,25,27]. The overall effect was low and non-significant (*g* = 0.17; 95% CI = −0.21 to 0.55; *p* = 0.39; Figure 6). True heterogeneity across the studies was small (I^2^ = 18.73%; Q = 3.08; df = 3; *p* = 0.38), the funnel plot was symmetrical (SE = 1.56; Supplementary Materials, Figure S1).

## 4. Discussion

The present systematic review aimed to profile the TR intervention approach for CHF and to test its effect on exercise capacity and quality of life compared to the NI and CI with a meta-analysis. Among the 11 RCTs included in the review, the majority (81%) presented good-to-high internal and external validity based on the TESTEX criteria. People with CHF participating in the trials were mainly old adult males with reduced left ejection fraction and heterogeneous etiology, and prevalently ischemic.

### 4.1. Telerehabilitation Intervention Descriptors

Both center- and home-based rehabilitation statements highlighted the value of a multidisciplinary approach to reducing the risk of cardiovascular exacerbations by acting together on the increment of physical activity, healthy eating, medication adherence, stress management, and smoking cessation [3,29]. Of the studies included in the review, little less than half of the studies proposed a unidimensional treatment focused solely on physical activity without a comprehensive approach. Considering the TR programs for CHF reported in the literature, a multidimensional approach’s significance has not always been considered. The duration of the TR intervention varied among studies, with a minimum of 8 to a maximum of 12 weeks, in line with the recommendations for home-based interventions [3]. Also the treatment intensity was set according to the HF home-based rehabilitation statement [3], based on a heart rate reserve ranging from 40 to 80%. Concerning the type of intervention, the trials were homogenous in providing mostly individual sessions (81%). Instead, the trials included presented a heterogeneous treatment dose in terms of frequency, ranging from 2 to 5 weekly sessions, with a range span similar to the recommended home-based span of 3–5 weekly sessions, and the regular center-based program of 1–3 weekly sessions [3].

### 4.2. Telerehabilitation Technology and Actions

Regarding the technology used to perform the TR, the use of healthcare platforms and monitoring devices were frequent, while only 27% of works included digital contents. Based on the maturity of the TR model, a complex and comprehensive kit would be necessary to handle the bidirectional communication between patients and clinicians in an asynchronous modality. Globally, technology adopted allowed a TR approach presenting asynchronous models, monitoring and assessment, decision, and offline feedback during the treatment period. This scenario revealed an established approach favoring the supervision, adaptability, and personalization of rehabilitation at a distance for the period of the treatment [11,12]. The flexibility of asynchronous TR may be supported by a mature and complex facility able to integrate all technological functionalities (such as data storage from different monitoring devices and rehabilitation performance recording) in a structured platform system. However, the utilization of digital contents for rehabilitation activities has been reported with beneficial effects on patient engagement with a critical role when the treatment is performed at a distance [30], and their role in cardiovascular rehabilitation may be relevant.

### 4.3. Telerehabilitation Safety and Adherence

Safety is a critical issue for CHF, especially for higher-risk patients who may benefit from home-based and TR treatment. Both safety and adherence were under-investigated in the studies. However, the results favored the TR approach by reporting minimal adverse events and high adherence (86%) to the treatment. These results suggest TR for CHF patients, mostly presenting an asynchronous model, as a suitable and safe approach. The safety of the TR asynchronous approach is an important issue. In fact, carrying the TR in an asynchronous way may impact care process sustainability, with positive effects on patients’ care delivered at home in the individual’s social context, caregivers’ time burdening with reduced distance barriers, and clinicians’ activities, which are efficient and capable to manage more patients at a distance. More studies focused on the safety of TR are needed for a more accurate and powered analysis. Concerning treatment adherence, the flexibility of asynchronous TR would have impacted the program’s attendance rate with positive effects on the patient’s everyday routine. The feeling of being actively engaged in the care process within the context of an asynchronous TR may have affected the participation of patients.

### 4.4. Telerehabilitation Effect

The eight studies included in the meta-analyses showed no accord in terms of outcome measures tested, with only a few of the works testing the effect of TR on mortality, anxiety and depression, cognition, sleep, cardiovascular risk, balance, walking speed, strength, and physical activity. The most frequent outcomes were functional capacity and quality of life. Concerning the functional capacity, the meta-analysis results supported a superiority effect of TR on NI and a non-inferiority effect of TR on CI in VO_2_ peak intake. Moreover, a non-inferiority effect of TR on NI and CI was observed in the 6MWD. In particular, the effect of TR on NI intervention on VO_2_ peak intake was large, in line with the results of a recent meta-analysis on TR for people with HF [13]. Therefore, TR benefits oxygen intake capacity also in the chronic phases of HF. Instead, contrary to the results of Cavalheiro et al. [13], the effect of TR on NI was not significant on the 6MWD. Also, the TR effect on the quality of life was comparable in all three conditions, TR, NI, and CI, in people with CHF. This result is in contrast with the previous meta-analysis with HF [13], registering a consistent improvement in quality of life after TR at home. However, the studies selected for the present review included only patients in chronic conditions. It is well known that working on quality of life in chronic disability is a critical issue. Also, the lack of TR effect on quality of life may be linked with two aspects. First, the type of TR intervention was mainly motor, and only just over half of the studies provided a comprehensive and multidimensional treatment focused on other components in addition to physical activity, aiming to assure a broad-spectrum benefit in the everyday life of the person. Second, it would be plausible to assume that TR for CHF, as currently conceived, does not contribute to the increment in quality of life. Especially an engagement and educational component should be integrated into the care program at a distance as a nodal core of the treatment allowing a patient’s sense-making experience for people with chronic conditions. In particular, the multidisciplinary, patient-centered management of CHF patients with an educational approach aligns with the Chronic Care Models for the continuity of care [31,32].

## 5. Conclusions

In conclusion, TR is an additional health strategy for people in need of rehabilitation, such as people with CHF. Current evidence suggests TR is a safe treatment, with minimal adverse events, promotes adherence, and improves functional capacity compared to NI. However, TR for people with CHF shows a heterogeneous scenario in terms of treatment type and dose. Future works may focus on the standardization of TR protocols for CHF, especially in terms of the type and dose of the intervention. The enhanced maturity of the TR system will allow the strengthening of the communication modality and the information of the recorded data. Finally, the integration of an engagement component into the TR model for HF remains a critical issue that needs to be addressed in future works.

## Figures and Tables

**Table 1:** TESTEX score.

Studies	1	2	3	4	5	6	7	8	9	10	11	12	Tot
	Eligibility	Randomization	Allocation	Groups Similarity at Baseline	Assessor Blinding	Outcome Measures	Intention-to-Treat	Between Group Statistical Comparison	Point Measures and Measures of Variability	Activity Monitoring in Control Group	Exercise Intensity Remained Constant	Exercise Volume and Energy Expenditure	
[18]	1	1	1	1	0	2	0	1	1	0	1	1	10
[19]	1	1	1	1	1	3	1	2	1	1	1	1	15
[20]	1	1	0	1	0	3	1	2	1	0	1	1	12
[21]	1	1	1	1	1	1	0	2	1	1	1	1	12
[22]	1	0	0	0	0	3	0	2	1	0	1	1	9
[23]	0	0	0	0	0	0	0	1	1	0	1	1	4
[24]	1	1	1	0	0	2	1	1	1	0	1	1	10
[25]	1	0	0	1	0	2	0	1	1	1	1	1	9
[26]	1	0	0	1	1	2	0	1	0	0	1	1	8
[27]	1	1	0	1	0	3	0	1	1	1	1	1	11
[28]	1	0	1	0	0	0	0	1	1	0	0	0	4

**Table 2:** Demographics and clinical characteristics of experimental and control groups of trials included in the systematic review. TR = Telerehabilitation; CI = conventional intervention; NI = no intervention.

Study	Group	Subjects [N]	Sex [N Male; Female]	Age (y) [M; SD]	HFLEF	HF Etiologies	VO_2_ [M; SD]
[18]	TR	20	18; 2	65.50; -	5% Medium; 20% Medium-Reduced; 75% Reduced	55% Ischemic; 45% non-ischemic	-
	CI	20	16; 4	71.20; -	15% Medium; 35% Medium-Reduced; 50% Reduced	75% Ischemic; 25% non-ischemic	-
	NI	20	17; 3	61.40; -	20% Medium; 30% Medium-Reduced; 50% Severe	65% Ischemic; 35% non-ischemic	-
[19]	TR	24	19; 5	68.00; 14.00	Reduced (LVEF% = 36.00 ± 16.00)	58% Ischemic; 4% valvular; 17% idiopathic dilated cardiomyopathy; 13% HF with preserved EF	-
	CI	29	21; 8	67.00; 11.00	Reduced (LVEF% = 35.00 ± 17.00)	52% Ischemic; 3% valvular; 21% idiopathic dilated cardiomyopathy; 7% HF with preserved EF	-
[20]	TR	15	9; 6	59.80; 10.00	Medium (LVEF% = 44.50 ± 17.30)	6% Ischemic; 43% dilated cardiomyopathy; 14% sarcoidosis; 7% amyloidosis; 7% hypertensive; 29% other	-
	CI	15	7; 8	67.70; 8.90	Reduced (LVEF% = 39.90 ± 17.80)	20% Ischemic; 25% dilated cardiomyopathy; 25% sarcoidosis; 8% amyloidosis; 17% hypertrophic cardiomyopathy; 25% other	-
[21]	TR	49	28; 21	-	Reduced (LVEF% = 34.03 ± 6.64)	61.2% Ischemic; 14.3% valvular; 14.3% idiopathic cardiomyopathy; 10.2% other	-
	NI	49	30; 19	-	Reduced (LVEF% = 34.07 ± 6.66)	59.2% Ischemic; 18.4% valvular; 14.3%; idiopathic cardiomyopathy; 8.1% other	-
[22]	TR	75	64; 11	54.40; 10.90	Reduced (LVEF% = 30.00 ± 8.00)	66.7% Ischemic; 33.3% non-ischemic	16.10; 4.00
	NI	32	31; 1	62.10; 12.50	Reduced (LVEF% = 34.00 ± 6.00)	84.4% Ischemic; 15.6% non-ischemic	17.40; 3.30
[23]	TR	36	31; 5	52.60; 10.12	Reduced (LVEF% = 32.00 ± 7.00)	69.4% Ischemic; 30.6% non-ischemic	16.98; 4.02
	NI	15	15; 0	61.40; 13.20	Reduced (LVEF% = 33.00 ± 8.00)	80.0% Ischemic; 20.0% non-ischemic	17.90; 3.61
[24]	TR	425	377; 48	62.60; 10.80	Reduced (LVEF% = 31.00 ± 7.00)	66.1% Ischemic; 33.9% non-ischemic	16.9; 6.0
	NI	425	376; 49	62.20; 10.20	Reduced (LVEF% = 30.00 ± 7.00)	64.5% Ischemic; 35.5% non-ischemic	16.6; 6.0
[25]	TR	75	64; 11	56.40; 10.90	Reduced (LVEF% = 30.20 ± 8.20)	73.3% Ischemic; 26.7% non-ischemic	17.80; 4.10
	CI	56	53; 3	60.50; 8.80	Reduced (LVEF% = 30.80 ± 6.70)	85.7% Ischemic; 14.3% non-ischemic	17.90; 4.40
[26]	TR	20	15; 5	57.80; 8.10	Reduced (LVEF% = 27.80 ± 8.80)	-	10.10; 3.10
	NI	20	14; 6	58.90; 6.90	Reduced (LVEF% = 26.00 ± 8.30)	-	10.10; 2.80
[27]	TR_1_	17	-	51.76; 9.83	Reduced (LVEF% = 29.59 ± 6.61)		15.40;2.70
	TR_2_	17	-	50.82; 9.45	Reduced (LVEF% = 31.00 ± 5.02)	-	15.60; 2.70
	NI	11	-	53.00; 8.19	Reduced (LVEF% = 31.55 ± 5.77)	-	15.70; 3.00
[28]	TR	26	25; 1	60.00; 8.50	Reduced (LVEF% < 35)	42.6% Ischemic; 34.5% Other, 23.1% Unknown	13.00; 2.30
	NI	26	22; 4	65.10; 8.20		50.0% Ischemic; 46.2% Other, 3.8% Unknown	10.70; 3.20

**Table 3:** Characteristics of TR program. HR = heart rate; I = individual; G = group; W = week; A = asynchronous; S = synchronous; VO_2_ = max oxygen consumption; EHO = event Holter.

Study	FITT Descriptors	TR APPROACH				Technology
		Model	Monitoring/ Assessment	Decision	Feedback	
[18]	Frequency: 2 sessions/W for 8 W Intensity: 40–60% HR reserve Time: 60 min Type: I, M (educational + aerobic exercise training)	A	Y	Y	Offline	Device: HR monitor Digital content
[19]	Frequency: 2 sessions/W for 12 W Intensity: 9–13 score at perceived exertion scale Time: - Type: G, M (educational + exercise training)	S	Y	Y	Online	Platform: videoconference platform Device: automatic sphygmomanometer, finger pulse oximeter Digital content
[20]	Frequency: 5–7 sessions/W for 15 W Intensity: 11–13 score at Borg scale Time: 30–40 min Type: I, M (education + stretching/resistance exercise training)	A	Y	Y	Online/Offline	Device: Fitbit + smartphone Digital content
[21]	Frequency: 3–5 sessions/W for 8 W Intensity: 40–70% HR reserve + HR at rest Time: 20–30 min Type: I + G, M (education + aerobic/resistance exercise training)	S	Y	Y	Online	Platform: QQ + WeChat + videoconference platform
[25]	Frequency: 3 sessions/W for 8 W Intensity: 40–70% HR reserve + 11 score at Borg scale Time: 45 min Type: I, M (education + psychological support + aerobic/resistance exercise training)	A	Y	Y	Offline	Platform: Data transmission survey Device: EHO 3 device (electrocardiogram) + mobile phone
[22]	Frequency: 5 sessions/W for 8 W Intensity: 40–70% HR reserve + Borg scale Time: - Type: I, U (aerobic/resistance exercise training)	A	Y	N	Offline	Platform: Data transmission survey Device: EHO 3 device (electrocardiogram) + mobile phone + blood pressure measuring + weighting machine
[23]	Frequency: 5 sessions/W for 8 W Intensity: 40–70% HR reserve + functional capacity at CPET Time: 10–45 min Type: I, M (aerobic/resistance exercise training + respiratory exercise training)	A	Y	Y	Offline	Platform: Data transmission survey Device: EHO 3 device (electrocardiogram) + mobile phone
[24]	Frequency: 5 sessions/W for 8 W Intensity: 40–70% HR reserve + 5–10 repetitions for resistance/strength exercises + 30–60% of Pi for respiratory exercises Time: - Type: I, M (aerobic/resistance/strength exercise training + respiratory exercise training)	A	Y	Y	Offline	Platform: Data transmission survey + monitoring platform Device: EHO 3 device (electrocardiogram) + mobile phone + blood pressure device + weighting machine
[26]	Frequency: 12 W Intensity: 80–85% VO_2_ peak followed by 40–50% VO_2_ peak + moderate intensity for resistance exercises Time: - Type: I, U (aerobic/resistance exercise training)	A	Y	Y	Offline	Device: HR monitor + pedometer
[27]	Frequency: 12 W Intensity: HR anaerobic threshold + 30–40% repetition maximum for strength exercises Time: - Type: I, U (aerobic/strength exercise training)	A	Y	Y	Offline	Device: HR monitor + free weights
[28]	Frequency: 5 sessions/W for 8 W Intensity: - Time: - Type: I, M (respiratory exercise training + strength/range-of-motion/isometric exercise training)	A	Y	Y	Offline	Platform: Data transmission survey Device: Electrocardiogram recording monitor

**Table 4:** Description of the outcome measures of the included studies *(n* = 11). VO_2_ = max oxygen consumption; NYHA = New York Heart Association; PER-BASED = performance-based; PR-BASED = patient-reported based.

Outcome	Domain	Subdomain	Tool	Per-Based	Pr-Based	Study
Medical-Benefit	Functional Capacity	Exercise capacity	VO_2_ peak	x		[22,23,24,25,26,27,28]
			Anaerobic threshold	x		[27,28]
			Exercise tolerance	x		[28]
			Shuttle walk test	x		[18]
			6-min walk distance	x		[19,20,21,22,24,25,26,28]
			Short physical performance battery	x		[20]
		Physical activity	activPAL ^TM^	x		[18]
		Strength	dynamometer	x		[19,27]
		Walking speed	10 m walk test	x		[19]
		Balance	Balance outcome measure for elder rehabilitation	x		[19]
		Sleep	Polysomnography	x		[27]
		Heart function	Heart rate at rest	x		[21,25]
			Heart rate variability/turbulence	x		[23,24]
			LVEF	x		[21,22,23]
			NYHA classification	x		[21,25]
			Echocardiography	x		[28]
			Brain natriuretic peptide	x		[20]
			Kansas City Cardiomyopathy Questionnaire		x	[20]
		Urinary function	Revised Urinary Incontinence Scale		x	[19]
		Frailty	Kihon checklist	x		[20]
	Participation	Quality of life	SF-36		x	[22,24,25]
			EuroQoL five-dimensional		x	[19]
			The Minnesota Living with Heart Failure Questionnaire		x	[19,21,26,27]
			Nottingham Health Profile		x	[28]
			EuroQol five-dimensional		x	[19]
		Mood	Hospital anxiety and depression scale		x	[21]
			Beck depression inventory		x	[28]
	Mortality					[24,25]
Patient-Relevant	Adherence		Participation rate	x		[19,20]
			Percentage of patients who carried out the training	x		[22,24,25,26]
	Safety		Adverse events		x	[19,20,24,25]
	Satisfaction		Client satisfaction questionnaire		x	[19]
			12-item *ad-hoc* questionnaire		x	[22]

## Data Availability

The protocol was registered in the International Prospective Register of Systematic Reviews (PROSPERO): registration ID CRD42022316822.

## Supplementary Materials

The following supporting information can be downloaded at: https://www.mdpi.com/article/10.3390/jcm12010064/s1, Table S1: Inclusion and exclusion criteria of the selected papers; Figure S1: Meta-analysis funnel plots.
