# Supplementary material for: Telerehabilitation Approaches for People with Chronic Heart Failure: A Systematic Review and Meta-Analysis
Source: J Clin Med. 2022 Dec 21;12(1):64. doi: 10.3390/jcm12010064 (PMC9820837; doi:10.3390/jcm12010064)
Supplement: Supplementary file 1 [file jcm-12-00064-s001.zip › jcm-2082717-supplementary.pdf]

Supplementary Material

Telerehabilitation Approaches for People with Chronic Heart Failure: A Systematic Review and Meta-Analysis

Sara Isernia [1], Chiara Pagliari [1,*], Nuccia Morici [1], Anastasia Toccafondi [1], Paolo Innocente Banfi [1], Federica Rossetto [1], Francesca Borgnis [1], Monica Tavanelli [1], Lorenzo Brambilla [2], Francesca Baglio [1] and on behalf of the CPTM Group †

[1] IRCCS Fondazione Don Carlo Gnocchi ONLUS, 20148 Milan, Italy
[2] IRCCS Fondazione Don Carlo Gnocchi ONLUS, 50143 Florence, Italy
* Correspondence: cpagliari@dongnocchi.it
† CPTM Group members are listed in the Acknowledgment section.

Table S1. Inclusion and exclusion criteria of the selected papers.

Study	Inclusion Criteria	Exclusion Criteria
[18]	Left ventricular systolic dysfunction; clinically stable for at least 1 month; optimized medication dosages	Significant ischaemic symptoms at low workloads; uncontrolled diabetes; acute systemic illness or fever; recent embolism; active pericarditis or myocarditis; moderate to severe aortic stenosis; regurgitant valvular heart disease requiring surgery; myocardial infarction within the past 3 onsets atrial fibrillation; decompensation; other comorbidities
[19]	Diagnosis of chronic heart failure; clinical heart failure symptoms; >18 years	Outlined by the Australian exercise guidelines criteria and no criteria (such as security parameters) to perform home-based telerehabilitation
[20]	Diagnosis of chronic heart failure (New York Heart	

	Association functional classification II–III) with physical frailty above pre-frailty.	Inability to perform 6MWT due to locomotor disorders or other reasons;<br>chronic kidney disease (estimated glomerular filtration rate < 30 mL/min/1.73 m2), pericardial disease, severe valvular disease, severe cognitive decline, 6MWD ≥ 550 m; ambulatory cardiological rehabilitation more than twice a week; readmission for acute exacerbation of HF within 1 month; absence of own smartphone; judged by the investigator as being ineligible for other reasons; BMI ≥ 25 kg/m2
[21]	Diagnosis of chronic heart failure for at least 3 months; >18 years; stable condition with a medical regimen for at least 4 weeks; skillful in using Wechat or QQ software via a smartphone; discharged to home; Chinese spoken	Myocardial infarction within the last month, unstable angina, uncontrolled hypertension, severe respiratory diseases, decompensated non-cardiac disease, malignancy, physical disability, mental disease
[22]	Left ventricular systolic heart failure diagnosed at least for 3 months; left ventricular ejection fraction <40% on echocardiography in class II or III according to the New York Heart Association (NYHA); clinically stable and stable medication regimen for at least 4 weeks; skillful in using a computer, tablet or smartphone	Unstable angina; acute coronary syndrome within the last month, coronary artery bypass grafting within the last 2 months, initiation of CRT-P or CRT-D within the last 6 months, implantation of a pacemaker and/or ICD within the last 6 weeks; symptomatic and/or exercise-induced cardiac arrhythmia; heart disease requiring surgical treatment; hypertrophic cardiomyopathy; severe pulmonary disease; uncontrolled hypertension; anemia; acute and/or decompensated noncardiac disease; physical disability or neurological problems; acute or chronic inflammatory disease; severe psychiatric disorder; denied to participate
[23]	Left ventricular systolic chronic heart failure diagnosed at least for 3 months; with a left ventricular ejection fraction (LVEF) < 40% on echocardiography in class II or III according to the New York Heart Association (NYHA); clinically stable and receiving an optimal and stable medication regimen for at least 4 weeks; no contraindications to cardiopulmonary training	Unstable angina; acute coronary syndrome within the last month, coronary artery bypass grafting within the last 2 months, initiation of CRT-P or CRT-D within the last 6 months, implantation of a pacemaker and/or ICD within the last 6 weeks; symptomatic and/or exercise-induced cardiac arrhythmia; heart disease requiring surgical treatment; hypertrophic cardiomyopathy; severe pulmonary disease; uncontrolled hypertension; anemia; acute and/or decompensated noncardiac disease; physical disability or neurological problems; acute or chronic inflammatory disease; severe psychiatric disorder; denied to participate

[24]	Left ventricular systolic heart failure have a LVEF≤40% on echocardiography in class I II or III according to the New York Heart Association (NYHA); hospitalization incident within 6 months; clinically stable; no contraindications to cardiopulmonary test; able to exercise using telerehabilitation	NYHA class IV; unstable medical conditions; history of the acute coronary syndrome within the last 40 days with LVEF ≤ 35%; percutaneous angioplasty within the last 2 weeks; coronary artery bypass grafting within the last 3 months; initiation of CRT; P or CRT; D or ICD or PM within the last six weeks; lack of ICD, CRT; P or CRT; D or PM therapy; intracardiac thrombus; rest heart rate >90/min; tachypnoea >20 breaths per minute; symptomatic and exercise; induced cardiac arrhythmia or conduction disturbances; acute myocarditis and/or pericarditis heart disease requiring surgical treatment; hypertrophic cardiomyopathy; severe pulmonary disease; uncontrolled hypertension; anemia
[25]	Left ventricular systolic heart failure diagnosed for 3 months with a left ventricular ejection fraction <40% on echocardiography in class II or III according to the New York Heart Association (NYHA); clinically stable and receiving an optimal and stable medication for at least 4 weeks; able to exercise using telerehabilitation	NYHA class I or IV; unstable angina; a history of an acute coronary syndrome within the last month, coronary artery bypass grafting within the last 2 months, or the initiation of cardiac resynchronization therapy (CRT) within the last year; symptomatic and/or exercise-induced cardiac arrhythmia or conduction disturbances; heart disease requiring surgical treatment; hypertrophic cardiomyopathy; severe pulmonary disease; uncontrolled hypertension; anemia; acute and/or decompensated non-cardiac disease; physical disability or neurological problems; acute or chronic inflammatory disease; cancer; severe psychiatric disorder; denied to participate
[26]	45-75 years; left ventricular systolic ejection fraction of <40%; VO2peak of <69% predicted for age; history of stable heart failure (NYHA Classes I-III); a stable dose of medication	Musculoskeletal limitation; pulmonary disorders that limit exercise; existing contraindications to exercise training; patients who are already involved in an exercise program
[27]	30–70 years; left ventricular ejection fraction <40% on echocardiography in class II or III according to the New York Heart Association (NYHA); peak VO2 <20	New York Heart Association10 Class IV, myocardial infarction or revascularization within the past 4 months, unstable angina, complex or symptomatic

	ml/kg/min; clinically stable and receiving an optimal and stable medication at least 2 months, b-blocker therapy, sleep apnoea and able to exercise telerehabilitation	ventricular arrhythmias, obstructive aortic or mitral valvular disease, hypertrophic cardiomyopathy, abnormal exercise testing, hypotension, pulmonary arterial pressure 50mmHg, COPD, physical or psychiatric disease
[28]	Diagnosed heart failure classified NYHA class III, left ventricle ejection fraction b35%, planned implantation of a CRT-D device, controlled hypertension, diabetes, and other metabolic disorders, capacity to perform treadmill exercise test, absence of complex arrhythmia	Acute or uncontrolled disorders other than CHF and severe mobility impairment; severe musculoskeletal conditions which preclude physical rehabilitation planned cardiac surgery or percutaneous coronary interventions, cardiac surgery or coronary angioplasty within last 3 months, acute coronary syndrome, stroke or TIA within last 6 months, venous thrombosis or pulmonary embolism in the past, significant valve, and pulmonary diseases

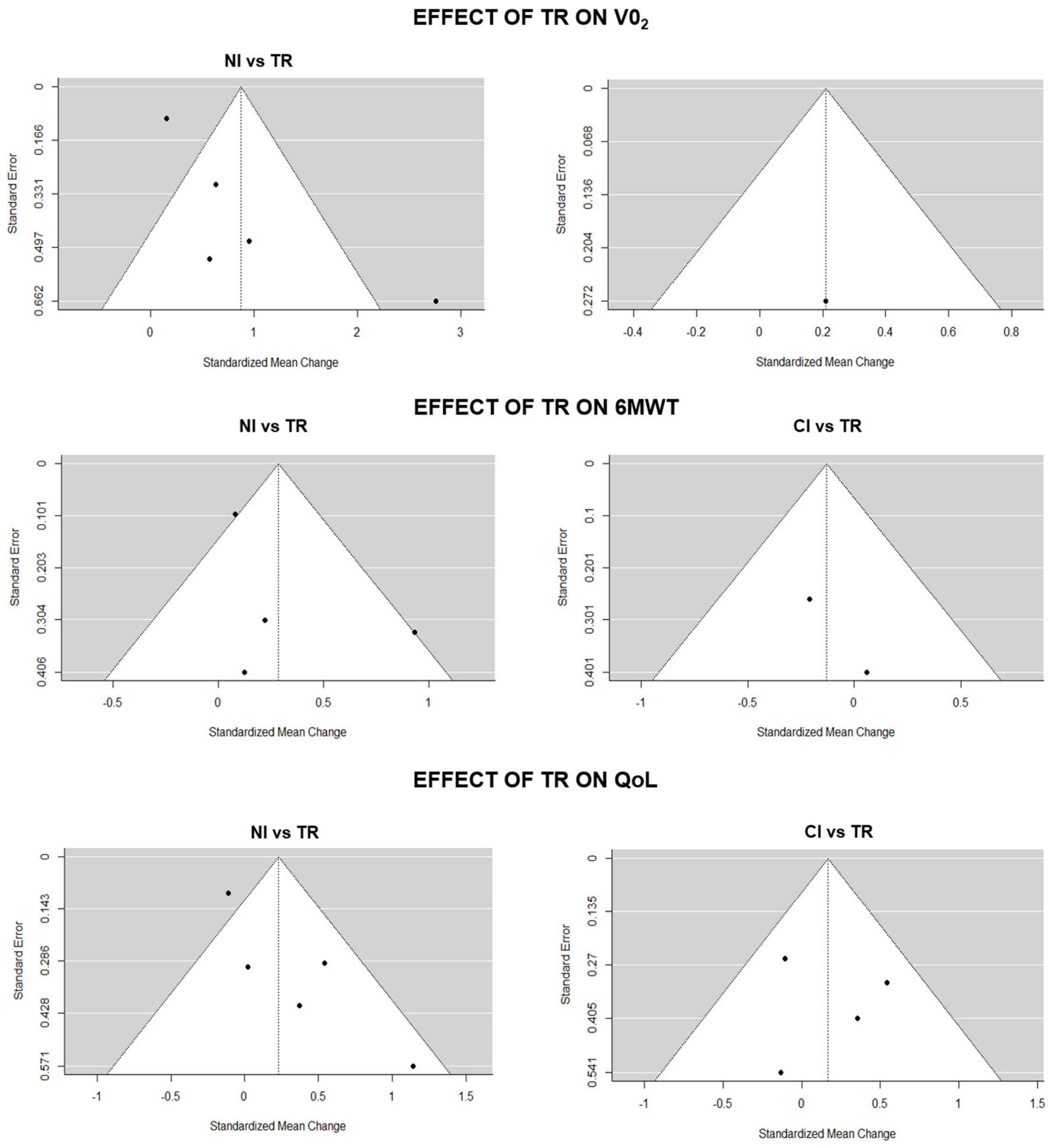

Figure S1. Meta-analysis funnel plots.
